# Evaluation of Two Configurations of Hydroxyapatite and Beta-Tricalcium Phosphate in Sinus Grafts with Simultaneous Implant Installation: An Experimental Study in Rabbits

**DOI:** 10.3390/dj11050121

**Published:** 2023-05-04

**Authors:** Ricardo Garcia Mureb Jacob, Ana Cláudia Ervolino da Silva, Liat Chaushu, Niklaus Peter Lang, Ciro Borges Duailibe de Deus, Daniele Botticelli, Idelmo Rangel Garcia Júnior

**Affiliations:** 1 Department of Diagnosis and Surgery, School of Dentistry, São Paulo State University (UNESP), Rua José Bonifácio 1193, Araçatuba 16015-050, SP, Brazil; ricardojacob@oralfit.com.br (R.G.M.J.); ana.ervolino@unesp.br (A.C.E.d.S.); ciro_duailibe@hotmail.com (C.B.D.d.D.); idelmo@foa.unesp.br (I.R.G.J.); 2 Department of Basic Sciences, School of Dentistry, São Paulo State University (UNESP), Rua José Bonifácio 1193, Araçatuba 16015-050, SP, Brazil; 3 Department of Periodontology and Implant Dentistry, The Maurice and Gabriela Goldschleger School of Dentistry, Tel Aviv University, Tel Aviv 69978, Israel; liat.chaushu@gmail.com; 4 School of Dental Medicine, University of Berne, CH-3010 Berne, Switzerland; 5 ARDEC Academy, I-47923 Rimini, Italy

**Keywords:** sinus floor augmentation, bone regeneration, rabbit, dental implant

## Abstract

Background: This study aimed to evaluate peri-implant bone formation in rabbits after sinus grafting mediated by hydroxyapatite and beta-tricalcium phosphate (HA + β-TCP) in granule or paste configurations, concomitant with immediate implant installation. Material & methods: Thirty-four rabbit maxillary sinuses were grafted with HA + β-TCP, half of which were applied in a granule and half in a paste composition. Implant placement was performed simultaneously. At 7 and 40 days postoperatively, the animals were euthanized, and samples were prepared for tomographic, microtomographic, histological, histometric (hematoxylin and eosin staining, HE), and immunohistochemical (labeling of transcription factor Runx-2 [RUNX2], vascular endothelial growth factor [VEGF], osteocalcin [OCN], and tartrate-resistant acid phosphatase [TRAP]) analysis. Implant removal torque was also measured. Results: On tomography, maintenance of sinus membrane integrity was observed in both the groups. Higher values of morphometric parameters evaluated by micro-CT were found in the “paste group” after seven days. At 40 days, there were no significant differences between the groups in most of the microtomographic parameters evaluated. In histological sections stained with HE, a higher percentage of newly formed bone was observed in the “granule group” after 40 days. Similar positive immunolabeling was observed for both RUNX2 and OCN in both the experimental groups. TRAP immunolabeling was similar in both groups as well. VEGF labeling increased in the “granule group”, indicating a higher osteoconductive potential in this biomaterial. Similar removal torque values were observed in both groups. Thus, the two HA + β-TCP configurations showed similar healing patterns of simultaneously installed implants adjacent to sinus floor elevation. However, significantly higher bone values were observed for the “granule configuration”. Conclusions: The HA + β-TCP granules and paste presentations showed favorable long-term healing results, with bone formation in similar quantities and quality adjacent to the implants.

## 1. Introduction

Dental implants are increasingly used for the rehabilitation of patients with one or more missing teeth. However, bone volume deficiency may be a negative factor when planning rehabilitation with implant-supported prostheses in the posterior maxilla, owing to the presence of the maxillary sinus, and hence, loss of alveolar bone height [1,2,3]. Such loss may be caused by atrophy after prolonged edentulism, resorption of alveolar bone in patients with periodontitis, pneumatization of sinus cavities [4,5,6], or a combination of these aspects.

Augmentation of the maxillary sinus prior to or concomitant with implant installation is one of the suggested treatments to promote an increase in bone volume in these regions. Bone grafting is a predictable and safe procedure [5].

Several materials have been used for sinus grafting and can be divided into four groups according to their origin: autogenous, allogenous, xenogenous, or synthetic bone grafting materials. These can be applied in single or combined applications [5,7,8].

Autogenous bone is considered as the “gold standard” for grafting biomaterials because of its osteogenic properties, osteoconductivity, and possible osteoinduction properties [5,9]. However, the use of autogenous bone is limited by restricted intraoral donor availability, intraoral and extraoral morbidity, the need for hospitalization, general anesthesia accompanied by postoperative pain, and high medical expenses in more advanced cases [5,10,11]. Allogeneic and xenogeneic bone have limitations, such as, e.g., the possibility of immunogenic rejection and transmission of infectious diseases [12].

Alloplastic grafting materials consist of synthetic substances such as polymers, calcium sulfate, hydroxyapatite, and calcium phosphates, or natural substances such as coral and algae-derived hydroxyapatite. Typically, these materials are regarded exclusively as osteoconductive and lack osteoinductive characteristics. Numerous studies have established their effectiveness in sinus augmentation when used alone or in combination with other grafting materials [13].

A biphasic calcium composite (BCC) composed of β-tricalcium phosphate and calcium sulfate has been proven in a clinical study to present clinical benefits in the treatment of periodontal defects such as autogenous bone [14]. Calcium sulfate (CaS), prepared in cementum or bead conformations, was used to fill bone defects produced in tibial metaphyses. Both conformations showed high biocompatibility and promoted new bone formation [15].

Ceramics based on biphasic calcium phosphate (BCP) have emerged as viable and well-accepted alternatives to autogenous bone owing to their similarity to bone matrix components, biocompatibility, and low immunogenicity. The combination of hydroxyapatite (HA) and β-tricalcium phosphate (β-TCP) in ceramics has important properties, as each component exhibits distinct physicochemical properties and biological advantages. β-TCP is biocompatible and highly porous [16], and is more soluble and resorbed faster than HA, which is more osteoconductive than β-TCP [12].

Synthetic HA and β-TCP have been tested in different compositions and physical configurations such as cements, particulates, metal implant coatings, and composites, with or without polymers, for use as matrices in tissue engineering and clinical applications [4,6,16]. In the Brazilian market, it is possible to find a formulation composed of HA and β-TCP in proportions of 60% and 40%, respectively, in granule and paste configurations.

In addition to the physical, chemical, and biological characteristics of bone grafts, another important factor in fixed implant-supported rehabilitation is time. Therefore, the sinus lift technique in humans, in which implants are installed concomitantly with the grafting procedure, may be preferred by clinicians to reduce treatment time [1,17].

The experimental model of sinus grafting in rabbits [18], associated with concomitant implant installation [3], has been widely used because of its bone remodeling properties that are similar to those of humans [18,19]. A comparison of healing in rabbit sinuses elevated with either paste or granules has already been performed [20], but without simultaneous implant placement. Hence, the aim of the present study was to evaluate the peri-implant bone formed after 7 and 40 days in rabbit sinuses grafted with hydroxyapatite and beta-tricalcium phosphate (HA + β-TCP) in a granule or paste configuration, concomitant with implant installation.

## 2. Materials and Methods

The study protocol was reviewed and approved by the Ethics Committee on Animal Use (CEUA) of Araçatuba Dental School (FOA-UNESP-FOA Process No. 506-2016).

### 2.1. Experimental Animals and Sample Calculation

Male New Zealand rabbits, approximately five months old and weighing 3–4 kg, were used. Throughout the experimental period, the animals were kept in identified cages in the animal house of FOA-UNESP, in an environment with a temperature between 22 °C and 24 °C, a controlled light cycle (12 h light and 12 h dark), and with the consumption of solid food and water ad libitum.

In the absence of data regarding the same materials used in the present study, data on new bone formation from a similar experiment performed on rabbits by the same research group were used [3]. By applying α = 0.05, a power of 0.8, and a calculated effect size of 2.34, the total sample size obtained was 4 for each group. An n value of 6 was considered sufficient to disclose the differences between the groups. The results of the sample calculation were obtained using G * Power 3.1 software (Franz Frau, University of Kiel, Germany).

### 2.2. Experimental Design

Twenty-nine rabbits underwent maxillary sinus grafting with a biomaterial composed of 60% HA and 40% -TCP-Osteosynt^®^ (EINCO Biomaterial Ltda, Belo Horizonte, MG, Brazil) and concomitant implant installation.

Twenty-four animals were randomly divided into two experimental groups with 12 animals per group, according to the biomaterial used: granules (20–40 mesh) or paste (100–200 mesh). The implants were randomly installed in one of the maxillary sinuses of the rabbits, and the contralateral side was filled with a randomly selected biomaterial and evaluated in another study. Tomographic, microtomographic, histological, and immunohistochemical analyses were performed.

In the remaining five animals of the twenty-nine included, both maxillary sinuses were randomly filled with the two biomaterials, and an implant was concomitantly installed in each sinus. An implant removal torque analysis was performed.

### 2.3. Surgical Procedure

The animals were weighed and sedated by an intramuscular (IM) injection of ketamine hydrochloride 1% (10 mg/kg) and xylazine hydrochloride 2% (5 mg/kg) (Francotar^®^, Virbac do Brasil Indústria e Comércio Ltda, Jurubatuba, SP, Brazil). Subsequently, the maxilla was trichotomized in the region corresponding to the maxillary sinuses, and antisepsis was performed using 1% aqueous polyvinylpyrrolidone solution. Local subcutaneous anesthesia was administered with infiltration of the anesthetic mepivacaine 2% (0.3 mL/kg) and vasoconstrictor epinephrine 1:100,000 for hemostatic and analgesic purposes in the immediate postoperative period.

**Figure 1:**
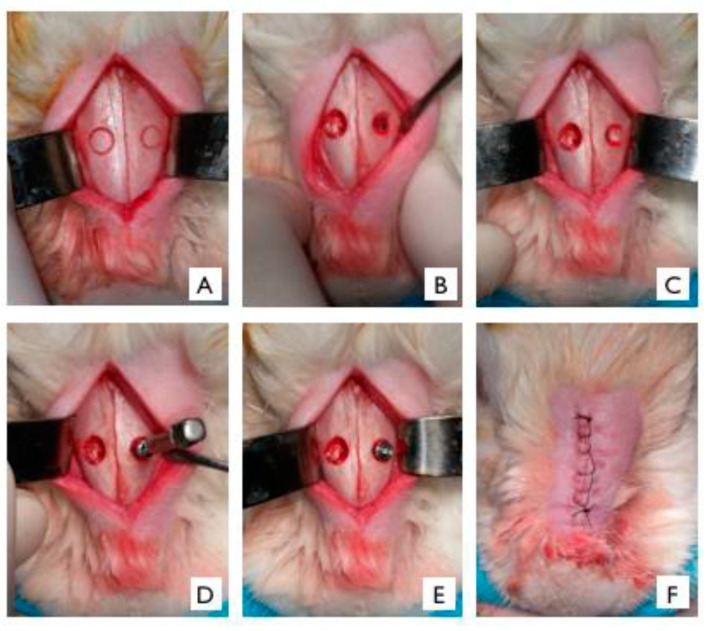
Operative sequence of sinus filling with HA + β-TCP in granules or paste and implant placement in the left maxillary sinus region of rabbits. (**A**) Demarcation of the maxillary buccal bone performed with a trephine drill 2 cm in front of the nasofrontal suture and 1 cm lateral to the midline. (**B**) Bone thinning with a diamond bur for detachment of the sinus membrane and exposure of the maxillary sinus interior. (**C**) Biomaterial applied inside the maxillary sinus. (**D**) Implant installation with the aid of a bidigital key. (**E**) Implant installed up to the prosthetic platform level (**F**) Soft tissue repositioning and suturing.

With the aid of a 15C blade (Feather Industries Ltd., Tokyo, Japan) mounted on a # 3 scalpel handle (Hu-Friedy, Leimen, Germany), a 5 cm linear incision was made in the midline of the nasal dorsum. The skin and periosteum were carefully detached and properly pulled apart with a Molt-type detacher (Molt Detacher, Quinelato, 18 cm, # 9, Rio Claro, SP, Brazil) to expose the nasal bone and naso-incisal suture. After exposure, the upper cortical bone of the maxilla of the animals was initially demarcated with a 5 mm trephine bur, the center being approximately 2 cm anteriorly from the nasofrontal suture and 1 cm lateral to the midline (Figure 1A), using a handpiece (Koncept 20:1, Kavo^®^ do Brasil, Joinvile, SC, Brazil) with a 20:1 reduction, and connected to an electric motor with controlled rotation (model BLM 600 plus, Driller^®^, Jaguaré, SP, Brazil) at a speed of 1500 rpm under copious irrigation with 0.9% saline solution. Then, inside the mark made with the trephine, the bone was delicately ground down with a 2.9 mm diameter diamond bur using a surgical handpiece (Koncept 1:2, Kavo^®^ do Brasil, Joinvile, SC, Brazil) connected to the electric motor until the entire sinus membrane was exposed. The sinus membrane was detached using curettes (Neodent^®^, Curitiba, PR, Brazil), exposing the sinus interior (Figure 1B) for the subsequent placement of the biomaterial (Figure 1C). In the “granule group”, a 2 g vial of HA + β-TCP, fractionated in 0.2 g per sinus, was used. In the “paste group”, a syringe containing 2 g of HA + β-TCP was used and fractionated in 0.2 g per sinus. The adaptation of the granules inside the maxillary sinus was performed using a Molt detacher, while the adaptation of the paste was performed with the syringe itself. In both groups, the biomaterial was applied concomitantly with the implant installation.

Standard commercial external hexagon implants, 3.6 mm in diameter and 6.5 mm in height (ATRO 3606, Implalife Biotechnology, Jales, SP, Brazil) were used. The implant was inserted using a bidigital key (Implalife Biotechnology, Jales, SP, Brazil) (Figure 1D,E) and stopped when the level of the prosthetic platform was flushed to the cortical bone layer. Nevertheless, despite the very low insertion torque used, defined as “Close to 0” [21], a stability of the implant was achieved.

The soft tissues were repositioned, co-adapted, and sutured in layers with 5-0 nylon threads (Figure 1F). All animals were medicated in the immediate postoperative period with a single IM dose of pentabiotic^®^ 1,200,000 units (0.1 mg/kg, Small Veterinary Pentabiotic, Fort Dodge Saúde Animal Ltda., Campinas, SP, Brazil) and tramadol hydrochloride (0.10 mL/kg–Tramal^®^, Schering-Plough S.A., Rio de Janeiro, RJ, Brazil).

**Figure 2:**
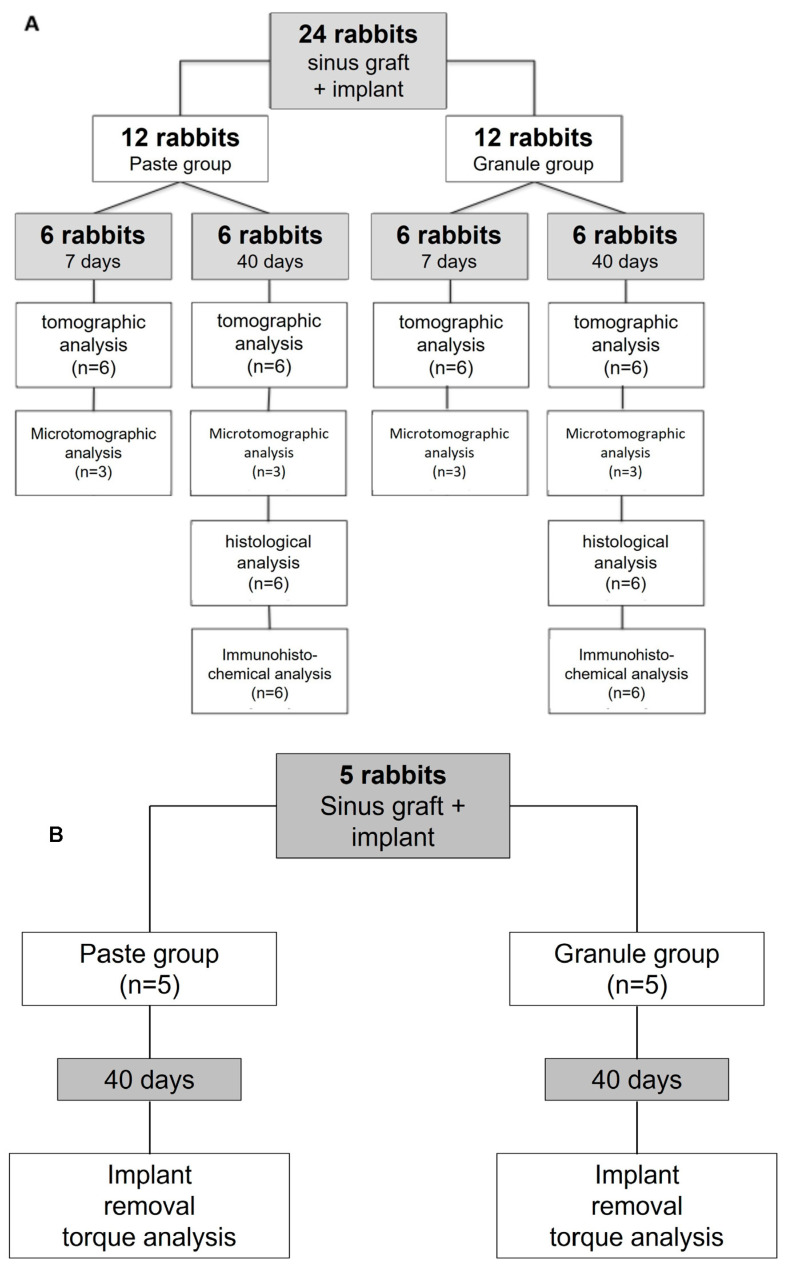
Flow charts of the distribution of animals in the experimental groups and their respective laboratory procedures on postoperative days 7 and 40. (**A**) Animals used for tomographic, microtomographic, histological, and immunohistochemical analyses. (**B**) Animals used for the analysis of implant removal torque. Please refer to the Section 2.2.

After sedation and injection of sodium pentobarbital 200 mg/kg, and after each period of healing (7 and 40 days), six rabbits in each group, “granule” and “paste”, were euthanized (Figure 2A). The remaining 5 rabbits out of the 29 were euthanized 40 days after surgery (Figure 2B).

### 2.4. Tomographic Analysis

Immediately after euthanasia, the naso-maxillary complex of all rabbits was removed in block, preserving the periosteum, and stored in 10% buffered formalin solution (Reagentes Analíticos^®^, Dinâmica Odonto-Hospitalar Ltda., Catanduva, SP, Brazil) in individual and identified fractions for 48 h. After fixation, the specimens were bathed in running water for 24 h and placed in 70% alcohol for tomographic and microtomographic analyses.

Twenty-four specimens (12 from each group in both experimental periods) were scanned using an i-CAT cone beam CT scanner (i-CAT Digital Imaging, Hatfield, PA, USA), and tomographic analysis was performed using the Dolphin Imaging software (Dolphin Imaging & Management Solutions, Chatsworth, CA, USA). Biomaterial and implant stability as well as sinus membrane integrity were analyzed in 1 mm-thick coronal sections.

### 2.5. Microtomographic Analysis

Three representative samples from each group were scanned using SkyScan^®^ 1272 microtomograph (Bruker MicroCT, Aartselaar, Belgium). The device was programmed with the following parameters: 90 kV voltage and 111 μA current, 0.5 mm aluminum and 0.038 mm copper filter, 0.5 rotation angle of 0.6 μm slices, and an image acquisition resolution of 2016 × 1344 pixels.

The maxillae were positioned on the sample holder of the equipment and fixed with utility wax for stabilization. The captured images were stored in TIFF format and then reconstructed from the implant apex, including the peri-implant region, using Nrecon software (SkyScan^®^, 2011; Version 1.6.6.0), with the following corrections: grade Gaussian noise attenuation, 7-pixel ring artifact, and 30% beam hardening, providing ideal images for analysis.

Using Data Viewer software (SkyScan^®^, Version 1.4.4 64-bit), the sequential set of images was emulated in the X-, Y-, and Z-axes, making it possible to observe the images in coronal X-Z, sagittal Z-Y, and axial X-Y orientations. Thus, the 3 images (coronal, sagittal, and axial) were aligned with regard to the long axis and the center of the implant, and the new positioning was used for analysis using CT Analyzer software (2003-11SkyScan^®^, 2012 BrukerMicroCT, version 1.12.4.0). Using this software, the tools for constructing the morphometric parameters of bone volume and bone-implant contact were chosen.

The region for analysis was defined from the limits of the graft in the coronal plane of the rabbit maxillary sinus and, in the sagittal plane, from the apical portion of the implant, not considering its conical part—that is, starting from the cylindrical portion and moving towards the coronal portion—until one-hundred 0.022 mm thick sections were obtained. The region of interest (ROI) was then determined dynamically in the previously defined region, passing through all images of the selected interval within the 100-slice limit.

The next step was the adaptive interpolation of the polygons, and in the binary selection tab, a maximum value of 57% and a minimum of 16% were determined for grayscale indexing, defining a better sharpness of the images in the histogram. Thus, it was possible to complete the preview of the morphometry of the volume to be studied and save the analyses in spreadsheet format for the OpenOffice.org 1.1 software (. csv).

For illustrative purposes, all samples were reconstructed and manipulated in three dimensions using the CTvox software (SkyScan^®^, Version 2.7), allowing visual analysis of the microtomographic reconstructions of the rabbit maxillary sinuses through transverse, coronal, and sagittal sections.

The following morphometric parameters were analyzed in both groups: bone volume (BV), bone volume percentage (BV/TV), trabecular thickness (Tb. Th), trabecular separation (Tb. Sp), trabecular number (Tb. N), total porosity [Po (tot)], and the intersection surface (iS) (Bouxsein et al., 2010).

### 2.6. Histological Preparation

After tomographic and microtomographic analyses, the samples were decalcified in 10% ethylenediaminetetraacetic acid (EDTA) and 20% sodium hydroxide and replaced every 7 days for approximately 25 weeks. The samples were then dehydrated in a graded ethanol series, cleared in xylene, infiltrated, and embedded in paraffin. The access was carefully carved into each paraffin block until the hexagon of the implant was fully exposed. A previously heated bidigital key was inserted into the hexagonal socket so that the heat transferred by contact allowed the implant to be unscrewed and removed with minimal damage to the peri-implant tissue. Finally, the blocks were again embedded in paraffin. Serial histological sections of the maxilla were obtained in the coronal plane. These were ground on a microtome (RM2235, Leica Biosystems Nussloch GmbH 2017, Nussloc, Germany) down to the region of interest to obtain 5 µm thick sections stained with hematoxylin-eosin (HE), for histological analysis of the peri-implant healing process and for histometric analysis of newly formed bone, residual graft, blood vessels, and connective tissue.

### 2.7. Histometric Evaluation

Maxillary sinus samples were digitally captured at 20× magnification (Leica DMLB, Heerbrugg, Switzerland) and blindly analyzed by an expert observer. The ImageJ software tool, which contained 391 points in an 8000-pixel model, was used to perform morphometry. The points corresponding to each evaluated variable were summed, and the total score for each variable was obtained. Three distinct areas of the peri-implant site at 40 days post-operation were determined and used for histometric analysis: the right lateral, left lateral, and sub-apical peri-implant sites. In each region, the percentage of newly formed bone, residual graft, blood vessels, and connective tissue was measured after 40 days [20].

### 2.8. Immunohistochemical Analysis

An additional histological section series was submitted to indirect immunoperoxidase for the detection of the transcription factor Runx-2 (RUNX2), vascular endothelial growth factor (VEGF), osteocalcin (OCN), and tartrate-resistant acid phosphatase (TRAP) to evaluate osteoblastic differentiation activity, vascular proliferation, bone mineralization stage, and osteoclastic activity, respectively. Immunohistochemical processing was performed only for samples corresponding to the 40-day experimental period.

The immunohistochemical processing began with the steps of deparaffinization (sections were kept in an oven for 20 min, followed by baths in citrisolv^®^ and baths in decreasing concentrations of alcohol) and ended with the hydration of sections immersed in phosphate-buffered saline (PBS; 0.01 M). Endogenous peroxidase activity was inhibited by hydrogen peroxide. Next, the slides underwent antigenic recovery with citrate phosphate buffer (pH 6.0) in moist heat. Endogenous biotin was blocked with skimmed milk for 20 min. Moreover, as a method for blocking non-specific staining, the primary antibody was prepared in a 1% phosphate buffer and bovine albumin solution.

The primary antibodies used were polyclonal antibodies, produced in goats, against RUNX2 (SC8566), VEGF (SC1881), OCN (SC18319) and TRAP (SC 30832) (Santa Cruz Biotechnology, Santa Cruz, CA, USA). The secondary antibody was a biotinylated anti-goat antibody produced in donkeys (Jackson ImmunoResearch Laboratories, West Grove, PA, USA). The reaction signal was amplified by incubation with avidin and biotin (ABC standard kit, Vector Laboratories, Burlingame, CA, USA), and the reaction was revealed using diaminobenzidine (Dako Laboratories, Santa Clara, CA, USA). At the end of the immunohistochemical reaction, counterstaining was performed using Harris hematoxylin. Subsequently, the slides were dehydrated and soaked in xylene, and coverslips were mounted for further analysis under an optical microscope (Nikon, Eclipse 80i, Shinagawa, Tokyo, Japan) with a 25× objective.

For each antibody used, expression of the proteins was evaluated by ordinal qualitative analysis through the attribution of different scores according to the number of cells and the extracellular matrix area immunolabeled during the healing process. The results are presented in tables showing the representative scores of the groups and the experimental periods analyzed. The evaluator was submitted to the Kappa test, and an index above 0.8 was obtained, which shows that the observed scores were consistent. The scores used were as follows: 0 (no labeling), 1 (light labeling, up to 25% of the area analyzed showed positive labeling for the protein analyzed), 2 (moderate labeling, up to 50% of the area analyzed showed positive labeling for the protein analyzed), and 3 (intense labeling, up to 75% of the area analyzed showed positive labeling for the protein analyzed).

### 2.9. Implant Removal Torque Analysis

Five samples from each group were used to measure the removal torque of the implants 40 days after installation. The implants were carefully removed using a counterclockwise force (removal torque or counter-torque) with a hand-held digital torque meter. The torque required to remove the implant from the bone was recorded (N · cm) and tabulated using an Excel spreadsheet.

### 2.10. Statistical Analysis

Histological data, microtomographic parameters, and implant removal torque were statistically analyzed using GraphPad Prism 7.04 software (La Jolla, CA, USA). Homoscedasticity was analyzed using the Shapiro–Wilk test to assess whether the values had a normal distribution. Microtomographic data were compared using analysis of variance and Tukey’s post-test, while histomorphometric and implant removal torque data were compared using a paired t-test. A significance level of 5% was considered statistically significant. The results of tomographic and immunohistochemical analyses are presented descriptively. The primary variable was new bone percentage, and the secondary variables were microtomographic parameters and implant removal torque.

## 3. Results

The surgical procedure was well tolerated, and all rabbits recovered quickly. No intensive care or force-feeding was required, and no clinical infection occurred.

### 3.1. Tomographic Analysis

**Figure 3:**
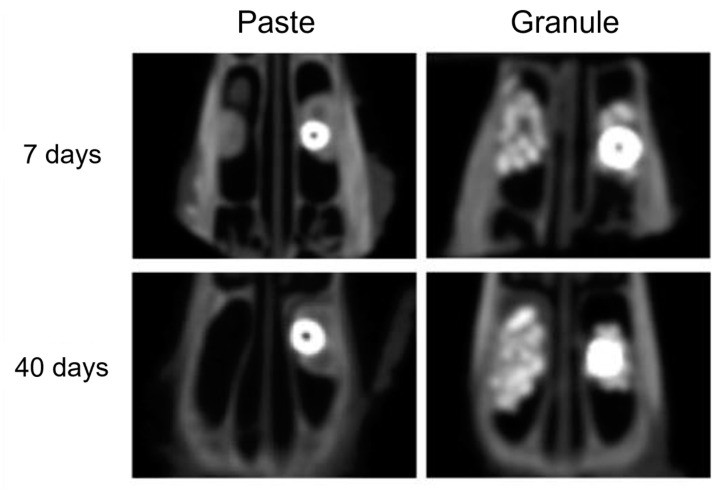
Tomographic images (axial sections) of the samples from the “paste” and “granule” groups after 7 and 40 days.

In all samples studied, there was no disruption or thickening of the sinus membrane of the rabbits after the surgical procedure. Consequently, the grafted biomaterials were contained within the maxillary sinuses with no leakage to the adjacent areas. Moreover, the implants remained stable 7 and 40 days after installation (Figure 3).

### 3.2. Microtomographic Analysis

**Figure 4:**
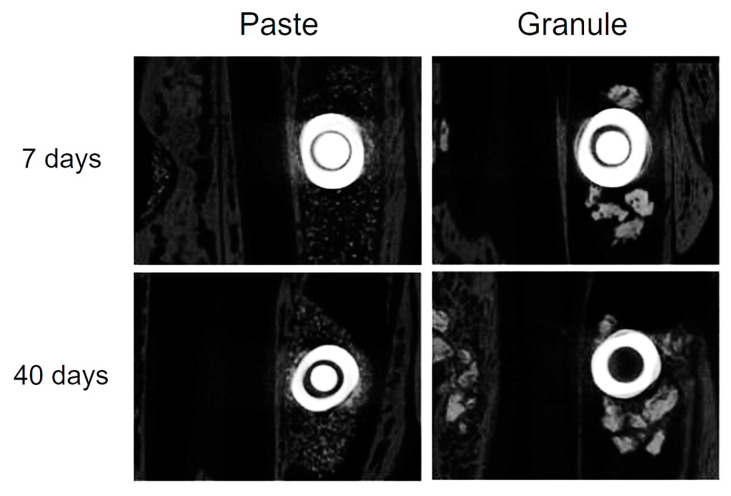
Microtomographic images (axial sections) of the samples from the “paste” and “granule” groups after 7 and 40 days.

**Figure 5:**
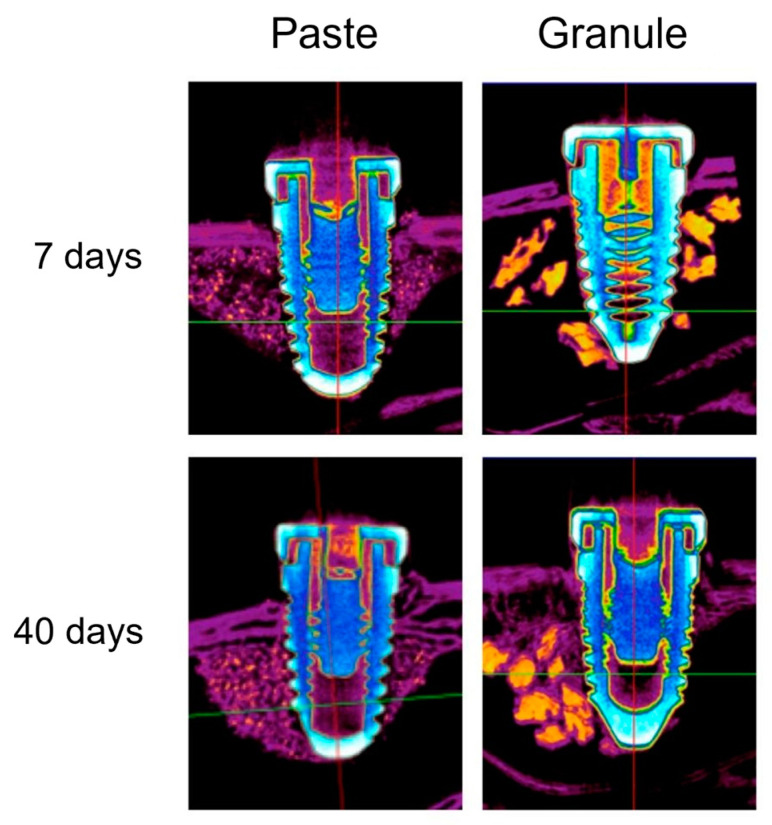
Microtomographic images (sagittal section-polarized images) of samples from the “paste” and “granule” groups at 7 and 40 days.

Figure 4 and Figure 5 show axial microtomographic and sagittal sections (polarized image), respectively, of rabbit maxillary sinuses filled with the biomaterial in paste or granule configurations at 7 and 40 days. At 7 days, the implants were in contact with the cortical bone, while most of the implant body was within the grafted maxillary sinus space in both groups. At 40 days, the newly formed bone had already occupied the maxillary sinus space and surrounded the implant body in both groups.

**Figure 6:**
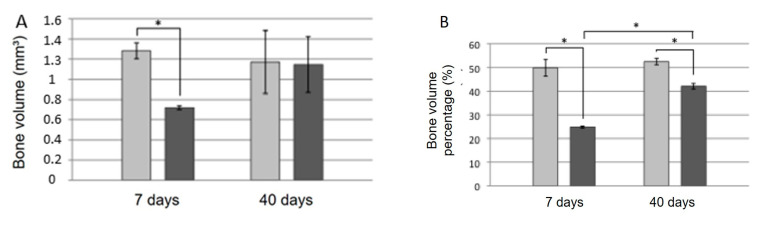

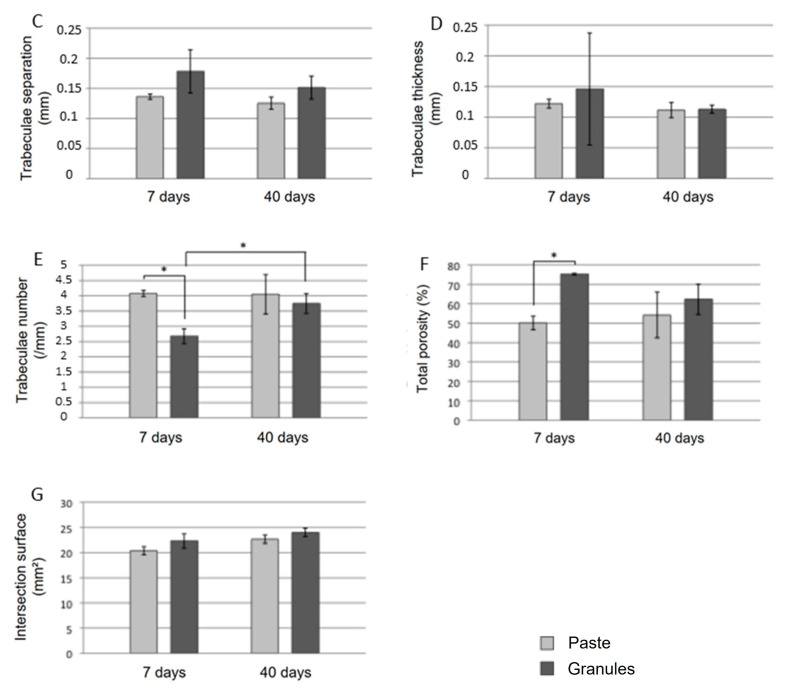
Morphometric parameters were quantified using computed microtomography in the “paste” and “granule” groups during the experimental periods of 7 and 40 days. (**A**) Bone volume (mm^3^) obtained by BV parameter analysis. (**B**) Bone volume percentage (%) obtained by BV/TV parameter analysis. (**C**) Bone trabecular thickness (mm) obtained by analyzing Tb.Th. (**D**) Number of trabeculae (/mm) obtained by analysis of parameter Tb.N. (**E**) Trabeculae separation (mm) obtained by analysis of parameter Tb.N. (**F**) Total porosity (%) obtained by analysis of parameter Po(tot). (**G**) Intersection surface (mm^2^) obtained from parameter iS analysis. *, *p* < 0.05.

The morphometric parameters quantified using microtomography are shown in Figure 6. The bone volume (*p* = 0.047), percentage of bone volume (*p* < 0.0001), and number of trabeculae (*p* = 0.0086) were significantly higher in the “paste group” than in the “granule group” at 7 days, whereas porosity was higher in the “granule group” during the same period (*p* = 0.0126). At 40 days, there were no significant differences between the groups for most of the microtomographic parameters studied, except for the percentage of bone, which was significantly higher in the “paste group” than in the “granule group” (*p* = 0.001).

Considering the same group at different experimental times, both the percentage of bone volume (*p* < 0.0001) and number of trabeculae (*p* = 0.0358) were significantly higher at 40 days than at 7 days in the “granule group.”

### 3.3. Histological Analysis

**Figure 7:**
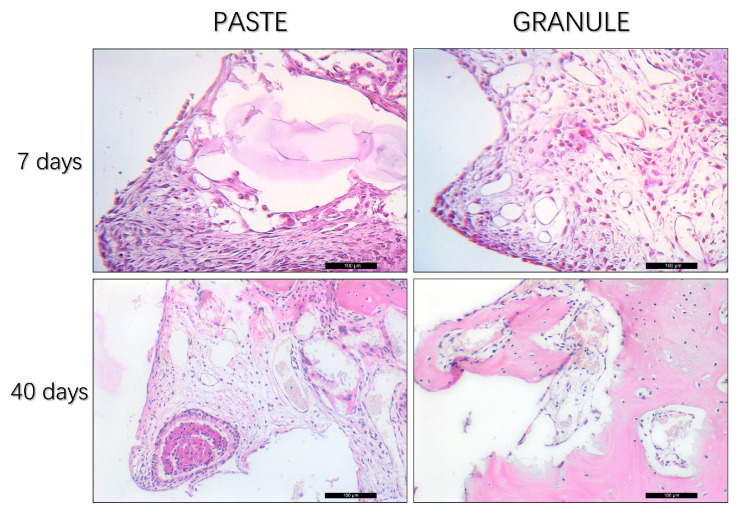
Photomicrographs of the rabbit maxillary sinus region at 7 and 40 days after filling with HA + β-TCP in “paste” and “granule” configurations, and concomitant implant installation stained in hematoxylin and eosin.

Seven days postoperatively, in the “paste group”, the presence of regular and homogeneous granules and abundant connective tissue rich in cells of infiltrated inflammatory tissue was noted. In the “granule” group, loose connective tissue was observed, which was richly vascularized and presented with giant cells (Figure 7).

At 40 days, a homogeneous residual granulometry pattern was noted in the “paste group”, without signs of inflammation, fine bone trabeculae, good cellular organization, and fewer blood vessels than in the “granule group”. In the latter group, a large number of irregular remaining granules and well-formed, organized bone tissue with thick trabeculae were observed (Figure 7).

### 3.4. Histometric Analysis

**Figure 8:**
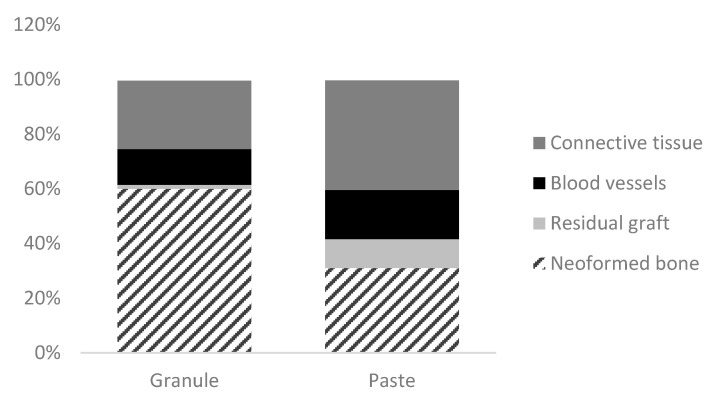
Representative graph of the percentage values of connective tissue, blood vessels, residual graft, and neoformed bone in the peri-implant region obtained by histometric analysis of the “granule” and “paste” groups.

A higher percentage of new bone was found in the “granule group” when compared to the “paste group” (60% vs. 31%, respectively; *p* < 0.001) (Figure 8; Table 1) while a higher content of connective tissue was found in the “paste group” than in the “granule group” (40% vs. 25%; *p* = 0.01). A greater amount of residual graft and blood vessels were found in the “paste group” than in the “granule group” (*p* > 0.05).

### 3.5. Immunohistochemical Analysis

Table 2 shows the representative immunolabeling scores for Runt-related transcription factor-2 (RUNX2), vascular endothelial growth factor (VEGF), osteocalcin (OCN), and tartrate-resistant acid phosphatase (TRAP) in the two groups at 40 days.

**Figure 9:**
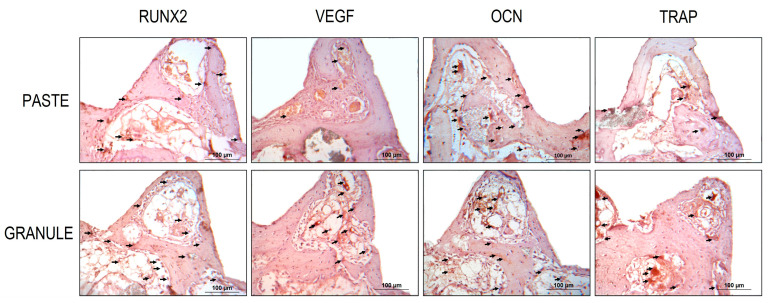
Photomicrographs of the maxillary sinus region of rabbits 40 days after filling with HA + β-TCP in the “paste and granule” configurations and concomitant implant installation, showing immunolabeling of RUNX2, VEGF, OCN, and TRAP proteins. The black arrows indicate examples of immunolabeling.

The transcription factor RUNX2 was positively labeled in pre-osteoblasts and young osteoblasts near the implant threads and near the biomaterial studied. Moderate to intense labeling (scores 2 and 3) was observed in the “granule group.” The same factor, evaluated in the “paste group” showed moderate marking (score 2). Bone tissue in bone remodeling activity was characteristic of most of the specimens evaluated in this group (Figure 9).

VEGF was positively labeled for osteoblasts and endothelial cells. It is important to highlight the labeling of blood vessels, especially in the “granule group”, where moderate labeling (score 2) was observed for this growth factor. In the “paste group”, light-to-moderate labeling (scores 1 and 2) was observed (Figure 9).

OCN, indicating the mineralized extracellular matrix and osteoblasts at a later stage of development, was moderately marked (score 2) in the “granule group” and moderately to intensely marked (scores 2 and 3) in the “paste group” (Figure 9).

The bone resorption marker enzyme TRAP was moderately marked (score 2) in the “granule group” and mild to moderately (scores 1 and 2) in the “paste group” (Figure 9).

### 3.6. Removal Torque Analysis

The mean values (±standard deviation) of the removal torque of the implants 40 days after installation were 9.4 ± 3.98 N.cm for the “granule group” and 9.4 ± 3.65 N.cm for the “paste group”, with no statistically significant difference between the groups (*p* > 0.05). The distribution of the values for the five animals in each group is shown in Table 3.

## 4. Discussion

This study evaluated peri-implant tissue formation after the sinus filling with paste and granules of a biomaterial composed of HA and β-TCP (60% and 40%, respectively), concomitant with implant installation in rabbits. The outcomes of the study were evaluated using tomographic, microtomographic, histological, immunohistochemical, and implant removal-torque analyses.

The experimental model used in the present experiment could resemble a sinus lift applying a crestal access, as occurs in humans, but using short implants [22]. However, in the rabbit model, dedicated instruments can be used to elevate the sinus membrane on the medial and lateral sinus walls, as in humans when using a lateral access.

The most common intraoperative complication during sinus lift procedures in humans is the perforation of the Schneiderian membrane [23,24,25], with an average occurrence of 19.5% (range, 5–56%) [26]. An association between membrane perforation and an increased implant failure rate has been reported [27], whereas adequately repaired perforations appear to have no effect on implant survival [28]. Nevertheless, another clinical study showed a negative correlation between the survival rate and perforation size [29].

Computed tomography is a non-invasive method for monitoring the progression, resolution, and anatomical variations associated with paranasal sinusitis, as it provides adequate images for the recognition of sinus changes, with a shorter acquisition time and lower cost than magnetic resonance imaging [30,31]. In the present study, no animals presented with perforations of the Schneiderian membrane, membrane thickening, or biomaterial inside the maxillary sinuses. The absence of secretions indicates the absence of inflammatory and/or infectious processes. Moreover, the implants were correctly positioned, their length and diameter were compatible with the size of the maxillary sinus, and they were fixed to the upper cortical bone. These factors, associated with the correct amount of biomaterial, favor the maintenance of membrane integrity and implant stability.

This outcome of the complete absence of perforations is not in agreement with the reported outcomes of similar studies. In these studies, it was shown that sharpened edges of the granules [32,33,34,35] and implant apexes and threads [33,36] in contact with sinus mucosae produced thinning and possibly perforations over time. These differences are due to the different analytical methods applied. While the evaluation made in the present study with cone beam computed tomography was performed on 1 mm-thick coronal sections, the analysis in the studies mentioned was performed under an optical microscope at high magnification. This allowed the discovery of progressive thinning of the sinus mucosa in contact with biomaterial granules, resulting in small perforations and ejection of granules outside the elevated space over time.

Computed microtomography has been used to evaluate volumetric and microarchitectural changes in newly formed bone and bone graft resorption over time [37,38]. Computerized microtomography for three-dimensional analysis of bone structures in vitro was first introduced by Feldkamp et al. [39]. The main advantage of this technique lies in the possibility of non-destructive and high-resolution analysis of porous materials, leaving the samples intact for further analysis, as opposed to histological analyses, which require cutting of the samples and two-dimensional evaluations. With its wide field of view, microtomography allows scanning of the entire region of interest in a sample [40]. However, owing to the high radiation exposure, the use of this technique has been limited to the evaluation of post-mortem mineralized tissues or in vivo studies with small animals.

In the present study, new bone formation appeared to start from the superior cortical bone of the sinus and progressed towards the center in the apical direction, corroborating the results of other studies [41,42,43]. By analyzing the morphometric parameters for each group separately, a significant increase in the percentage of bone volume and number of trabeculae was observed from 7 to 40 days in the “granule group”. In the “paste group”, bone volume, percentage of bone volume, and number of trabeculae at 7 days were very similar to the values obtained at 40 days and significantly higher than those observed in the “granule group” at that time. This seemingly contradictory result may indicate failure in the determination of these parameters, especially in the initial period after grafting the biomaterial in the paste configuration. A possible reason for the inability to estimate the formation of new bone in this group seems to be the similar radiodensity between the new bone and graft, both of which are composed predominantly of HA with greater particle compaction in the paste configuration than in the granule form. This difficulty in delimiting each tissue (residual graft and new bone) after sinus filling has also been reported in other human [44] and rabbit [45,46] studies.

In the histological analyses, a statistically significant higher amount of new bone was found in the “granule” compared to the “paste group”, as evidenced by the presence of mature bone tissue with thick trabeculae, conferring a more stable structure of the graft. On the other hand, a higher connective tissue percentage was found in the “granule” than in the “paste group”.

In the “granule group”, the presence of a large number of remnant granules surrounded by neo-formed bone was evident, which is similar to the results of other studies [20,47]. The rate of graft incorporation depends on the porosity of the material; the higher the macroporosity and microporosity, the greater the angiogenesis, leading to faster graft incorporation [48,49]. Thus, the composition and configuration of BCP may be used to control the inflammatory response that influences the course of wound healing [50].

It is worth mentioning that in both groups, it was possible to observe a large amount of residual material at 40 days. To evaluate the course of bone formation and possibly the total transformation of the material into bone in both configurations, studies with longer follow-up periods are required. In a rabbit study [20], a combination of hydroxyapatite (HA) and beta-tricalcium phosphate (TCP) at a ratio of 60:40 was used as granules or in a paste configuration. Similar to the present study, after 10 weeks of healing, higher proportions of new bone were observed in the “granule group” than in the “paste group”. However, approximately 38% and 49% of the residual graft was found, respectively.

The resorption rate of bone grafts is a very important characteristic that is related to the bioactivity of the material and the osteogenic effect. There are different means of degradation of calcium phosphate-based biomaterials, including resorption by osteoclast-like cells, chemical dissolution, and phagocytosis of very small particles by macrophages. HA is known to require a long interval for replacement with native bone due to its low replacement rate [51]. Studies suggest that the lower resorption of HA is due to the lower immune response mediated by T cells and inflammatory cytokines, consequently; there is less migration of foreign body giant cells and macrophages in the presence of this type of graft [52]. It is not known for sure if the higher amount of inflammatory cytokines is beneficial for the biological function of the material; however, there is evidence that the pure HA-based graft promotes less bone formation when compared to other allografts within a 12-week period [53]. Despite the lower rate of bone neoformation, the slower resorption of a biomaterial is an important feature for maintaining a framework to receive osteoblast precursor cells, contributing to long-term bone formation.

β-TCP is probably best known for its rapid resorption. Inflammatory cell-mediated biodegradation of β-TCP is more pronounced than that of HA. When it comes to biomaterials for bone regeneration, degradability is an important property that is directly related to bone regeneration capacity, which is why a large number of studies indicate better bone formation response to the presence of β-TCP [54,55,56,57]. The combination of HA and β-TCP is one of the main choices for bone regeneration when it comes to synthetic materials. HA has a relatively slow resorption rate but has greater mechanical strength. In contrast, β-TCP is more easily resorbed, but has lower mechanical strength. The resorption rate of this biomaterial can be controlled by varying the ratio of HA and β-TCP.

Immunohistochemical analysis showed that the detection of RUNX2, VEGF, OCN, and TRAP proteins in the two groups indicated that the configuration of the biomaterial stimulated osteoblastic differentiation (RUNX2), osteoconduction (VEGF), mineralization (OCN), and osteoclastic activity (TRAP). However, the “granule” configuration stimulated superior tissue responses with evident cell renewal (evidenced by RUNX2 staining), osteoconduction, moderate mineralization (which is accepted for the 40-day period), and resorption activity of the material (evidenced by TRAP staining). In the “paste group”, significant bone remodeling activity was observed in the tissue (different from the “granule group”, where the tissue was highly organized), suggesting that the tissue response was chronologically delayed compared to the “granule group”. In this group, there was also marked cellular renewal, as there was positive labeling for RUNX2, without an osteoconduction response as evidenced as in the “granule group”. TRAP was less frequently present than that in the “granule group”.

Despite subtle differences between the tissue responses of the two configurations of biomaterial at 7 days, the results of the analyses were generally similar at 40 days, which was supported by similar implant removal torque values in both groups. Implant removal torque is considered an objective method for evaluating osseointegration, and the values obtained in this test represent the interlocking between the implant and surrounding bone [58]. To our knowledge, this is the first study in which the implant removal torque was evaluated in the maxillary sinus of rabbits; hence, comparisons with other studies in which implants were installed in the tibia or femur of rabbits have low significance [59,60,61,62,63]. Thus, the long-term results were favorable with respect to both the quantity and quality of newly formed bone near implants installed concomitantly with sinus filling with the BCP-based biomaterial in both configurations.

In addition to evaluating the biological responses stimulated by the two configurations of the tested biomaterial, the handling properties are decisive in the choice of material for clinical application. In the present study, significant differences in these properties were observed between the two groups during the surgical stage. The configurations of HA + β-TCP in granules offer some resistance during placement in small areas, making it difficult to handle, whereas the paste configuration can be easily injected, even in hard-to-reach places. This is of great relevance, especially when there is a need for grafting in challenging sites, such as periodontal defects, where the material needs to be applied in a well-defined anatomical location and the tissue space needs to be supported by the filling material [8].

The size of the particles and the space between them in different configurations may also be important factors that affect the outcome of the procedure. Smaller particles may be resorbed more quickly and hence have a shorter lifetime as space maintainers [8]. Progressive resorption may jeopardize implant stability in maxillary sinus elevation, concomitant with implant installation [1]. Furthermore, the particles are compacted in a paste composition, which hindered angiogenesis. Therefore, this configuration is preferred exclusively for the filling of bone defects. In the granule configuration, the larger particles allow the permeability of the clot in the entire region of the graft, creating a favorable condition for angiogenesis and osteoconduction.

Another important factor to consider is the amount of material required to fill large cavities, such as maxillary sinuses and post-dental extraction alveoli [8]. Thus, it is possible to place a smaller amount of the biomaterial in the granules, resulting in the same volume as that of the paste configuration, which directly interferes with the cost of the procedure.

Antibiotics and analgesics were added as post-surgical therapy. This is generally the therapy that is also applied to humans, so that similar effects on healing might be expected.

As limitations of the present experiment, the model used should be considered in terms of dimensions, sinus mucosa width, and healing rate. The sinus mucosa in rabbits is thinner compared to humans [64,65,66]. Moreover, the rate of healing is faster in animals than in humans [67].

## 5. Conclusions

The HA + β-TCP granules and paste presentations showed favorable long-term healing results, with bone formation in similar quantities and quality adjacent to the implants.

From the results of each of the analyses performed in the present study, the following conclusions were drawn:

- Both configurations ensured the stability of the biomaterial, integrity of the sinus membrane, and the absence of inflammatory/infectious processes.
- Both configurations favored the formation of new bone from the superior cortical bone of the maxillary sinus, progressing towards the center in the apical direction. Owing to the radiodensity of each configuration, the “granule group” provided a better visualization of the progression of bone neoformation over time.
- Both configurations showed histological results consistent with the cellular events that occur during bone neoformation.
- Both configurations induced the activation of proteins important for repair, with the “granule” configuration showing superior osteoconductive potential, as evidenced by the higher VEGF immunolabeling.
- Implant stability was similar in both groups.

## Figures and Tables

**Table 1:** Histometric evaluation of new bone, soft tissue, residual graft, and vessels after 40 days of peri-implant repair in the rabbit sinus region. Data are reported in percentages (%).

	New Bone	Soft Tissue	Residual Graft	Vessels
Granule	60	25	2	13
Paste	31	40	11	18
*p* values Granule vs. Paste	*p* < 0.0001 *	*p* = 0.01 *	*p* = 0.09	*p* = 0.5

* *p* < 0.05.

**Table 2:** Representative immunolabeling scores for race-related transcription factor-2 (RUNX2), vascular endothelial growth factor (VEGF), osteocalcin (OCN), and tartrate-resistant acid phosphatase (TRAP) in the granule and paste groups after 40 days. Scores were classified as mild (1), moderate (2), or intense (3), according to the area of immunolabeling.

Markers	Paste (Scores)	Granules (Scores)
RUNX2	2	2–3
VEGF	1–2	2
OCN	2–3	2
TRAP	1–2	2

**Table 3:** Caption. Removal torque values of implants 40 days after installation.

Paste (N.cm)	Granules (N.cm)
14	16
9	10
9	6
4	7
11	8

## Data Availability

The data presented in this study are available on request to the corresponding author.
